# A gene-rich linkage map in the dioecious species *Actinidia chinensis *(kiwifruit) reveals putative X/Y sex-determining chromosomes

**DOI:** 10.1186/1471-2164-10-102

**Published:** 2009-03-10

**Authors:** Lena G Fraser, Gianna K Tsang, Paul M Datson, H Nihal De Silva, Catherine F Harvey, Geoffrey P Gill, Ross N Crowhurst, Mark A McNeilage

**Affiliations:** 1 The New Zealand Institute for Plant and Food Research Limited, Auckland 1142, New Zealand; 2 ViaLactia Biosciences (NZ) Ltd, Auckland 1031, New Zealand

## Abstract

**Background:**

The genus *Actinidia *(kiwifruit) consists of woody, scrambling vines, native to China, and only recently propagated as a commercial crop. All species described are dioecious, but the genetic mechanism for sex-determination is unknown, as is the genetic basis for many of the cluster of characteristics making up the unique fruit. It is, however, an important crop in the New Zealand economy, and a classical breeding program would benefit greatly by knowledge of the trait alleles carried by both female and male parents. The application of marker assisted selection (MAS) in seedling populations would also aid the accurate and efficient development of novel fruit types for the market.

**Results:**

Gene-rich female, male and consensus linkage maps of the diploid species *A. chinensis *have been constructed with 644 microsatellite markers. The maps consist of twenty-nine linkage groups corresponding to the haploid number n = 29. We found that sex-linked sequence characterized amplified region (SCAR) markers and the 'Flower-sex' phenotype consistently mapped to a single linkage group, in a subtelomeric region, in a section of inconsistent marker order. The region also contained markers of expressed genes, some of unknown function. Recombination, assessed by allelic distribution and marker order stability, was, in the remainder of the linkage group, in accordance with other linkage groups. Fully informative markers to other genes in this linkage group identified the comparative linkage group in the female map, where recombination ratios determining marker order were similar to the autosomes.

**Conclusion:**

We have created genetic linkage maps that define the 29 linkage groups of the haploid genome, and have revealed the position and extent of the sex-determining locus in *A. chinensis*. As all *Actinidia *species are dioecious, we suggest that the sex-determining loci of other *Actinidia *species will be similar to that region defined in our maps. As the extent of the non-recombining region is limited, our result supports the suggestion that the subtelomeric region of an autosome is in the early stages of developing the characteristics of a sex chromosome. The maps provide a reference of genetic information in *Actinidia *for use in genetic analysis and breeding programs.

## Background

New Zealand has a long history of interest in the genus *Actinidia*, being the country which commercialized the fruit of *Actinidia deliciosa *(A. Chev.) C.F. Liang *et *A.R. Ferguson var. *deliciosa*, as kiwifruit, and which recently released *Actinidia chinensis *Planch. var. *chinensis *'Hort16A', the gold-fleshed kiwifruit, as an alternative cultivar. While New Zealand was instrumental in bringing these fruits to commercial attention, the genus is native to China and neighbouring countries where more than 60 species are known. This germplasm is relatively unexplored in terms of horticultural development of new and novel cultivars and offers a huge range of fruit characters and 'eating attributes', and plants suited to a wide range of climatic conditions. The diversity of flavours, fragrances, colours, and health factors are also of interest in genomic studies, offering the possibility of defining chemical pathways and identifying gene function.

*Actinidia *species present challenges to research and breeding. All known species in the genus are dioecious. Female plants bear flowers that are hermaphroditic in appearance but produce only empty pollen grains, while male plants have flowers that are unisexual with numerous stamens surrounding a rudimentary pistil whose growth is suppressed before style elongation or ovule initiation. Full dioecism is shown by about 4% of seed plants, and a second group display a variety of sub-dioecious conditions [1]. Genetic studies have shown that dioecy has evolved many times in plants, and have demonstrated a variety of sex-determining systems [2,3]. In *Actinidia*, bulk segregant analysis with random amplified polymorphic DNA (RAPD) markers supported the hypothesis that sex-determining genes were localized in a pair of chromosomes that function like an XX/XY system with male heterogamety [4-6]. The small size (<1 μm) of the chromosomes has made cytological studies difficult with the techniques available, and sex-determining chromosomes have not been positively identified. He *et al. *[7] using an improved chromosome binding technique, analyzed the karyotypes of diploid *A. chinensis *at the primary differentiation stage and reported that the sex chromosomes could not be identified from karyotypes of somatic cells. However, when they examined the pachytene stage of pollen mother cell meiosis, all 29 pairs of homologous chromosomes of pistillate and staminate plants paired tightly, except for a pair of nucleolar (SAT-) chromosomes in staminate plants. The two SAT-chromosomes were similar in length and shape, but in staminate plants the SAT region, about 15% of the total nucleolar chromosomal length, did not pair. He *et al*. [7] suggested the SAT region of nucleolar chromosomes may be the region of sex determination. They also suggested that sex chromosomes were probably at an early stage of differentiation in *Actinidia*.

The DNA content of the 2C genome of *A. chinensis *measured by flow cytometry was reported to be 1.3 – 1.4 pg [8], which corresponds to about 1.3 × 10^9 ^bp per genome. The genus contains species that form a polyploid series from diploid to octoploid [9].

As kiwifruit is a relatively new crop, knowledge of its genetic make-up is limited, so the development of a comprehensive genetic map and the use of molecular markers have the potential to improve efficiency in breeding new cultivars. A map will also help to simplify genomic studies to identify and isolate genes. Genetic linkage maps based on the recombination values of molecular markers have been constructed in an increasing number of plants (tomato [10,11], rice [12], barley, [13], lotus [14], *Brassica *[15], cotton [16], grape [17]) and are proving valuable tools for plant breeding. The construction of a genetic map in an obligate outbreeding species, such as *A. chinensis*, is more complex than one derived from inbred or homozygous parents. Maps in outbreeding species have been developed by utilising the two-way pseudo-testcross procedure [18,19], where the mapping population is the F_1 _progeny of a cross between unrelated, highly heterozygous individuals. Constructing the linkage map is complicated, as the two-way pseudo-test cross may segregate for up to four alleles at any locus, with one or both parents heterozygous at any given locus. The linkage phase of the markers will often be unknown, and can be different for the two parents, which can lead to inaccuracies in the estimation of recombination frequencies [20,21]. The recombination frequencies can, however, be separately estimated for each parent so that two maps are developed, and these maps can be integrated using markers that are heterozygous in both parents. Two low density linkage maps have been reported in *Actinidia*. Testolin *et al. *[22] used the progeny of an interspecific cross between *A. chinensis *and *A. callosa *to construct, at a LOD score ≥ 2.0, a female map of 203 loci over 38 linkage groups and a male map of 143 loci over 30 linkage groups.

While marker systems such as restriction fragment length polymorphisms (RFLPs), amplified fragment length polymorphisms (AFLPs), random amplified polymorphic DNA (RAPDs), or single nucleotide polymorphisms (SNPs) have been developed to facilitate genetic mapping and gene discovery, the marker system of choice in many plant species is microsatellites (simple sequence repeats or SSRs). Microsatellites are arrays of short tandem repeat motifs of 1 to 5 base pairs in length which are characterized by their abundance, their distribution in both non-coding and coding regions of eukaryotic genomes, reproducibility, Mendelian mode of inheritance and co-dominant nature [23]. They are recognised as highly informative genetic markers because of their inherent variability. This hypervariability is due to the high mutation rate within the nucleotide sequences of the microsatellites, and increases with increasing number of tandem repeats. In humans, heterozygosities generally exceed 0.5 and range as high as 0.9, with as many as 50 alleles per locus [24], and mutation rates, though variable among loci, exceed rates for non-microsatellite loci by up to four orders of magnitude [25,26]. Similar hypervariability within microsatellite loci has been reported for birds, insects and plants, and loci may be polymorphic even in species where low levels of genetic diversity make alternate marker systems less useful [27,28].

The time-consuming and expensive process of developing enriched genomic libraries and the subsequent sequencing and seeking of the simple sequence repeats is now often replaced with data mining of expressed sequence tag (EST) libraries to give a rapid, efficient and low-cost alternative for identifying microsatellites in plant species. Microsatellites have been found to occur regularly in ESTs [29]. The frequency of occurrence of microsatellites of suitable length (20 nucleotides or more) varied in five cereals examined from 1.5% for maize to 4.7% for rice [30]. This percentage would be sufficient to yield numerous markers from plant species in which large numbers of ESTs have been developed. In *Actinidia *the frequency of occurrence and level of polymorphism of EST-derived di-nucleotide microsatellites were sampled and found to be numerous in both the 5' and 3' ends of the genes represented, and highly polymorphic (93.5%) in the mapping population [31].

The construction of a single map for a cross in an outbreeding species, rather than two separate maps for the parents, depends on the availability of markers that are heterozygous in both parents. These markers form allelic bridges [19]. Dominant markers such as RAPDs or AFLPs are generally of very limited use in combining parental maps, therefore, when the homologous linkage groups of the parents of a mapping population are required to be integrated, co-dominant markers such as microsatellites or RFLPs are the markers of choice, and allow the construction of either separate parental maps, or an integrated map for the cross [21].

Here we present comprehensive genetic linkage maps of female and male informative markers mapped in a cross in the outbreeding species *A. chinensis*, and also an integrated map of the cross, achieved through the use of co-dominant microsatellite markers. The twenty nine linkage groups are defined, and the position of sex-determining loci identified. Genetic linkage maps in *Actinidia *have been developed to supply markers for breeding novel cultivars, to provide tools for comparative and quantitative trait mapping, and to investigate the evolution and function of genetic control mechanisms.

## Results

### Linkage map construction

**Figure 1:**
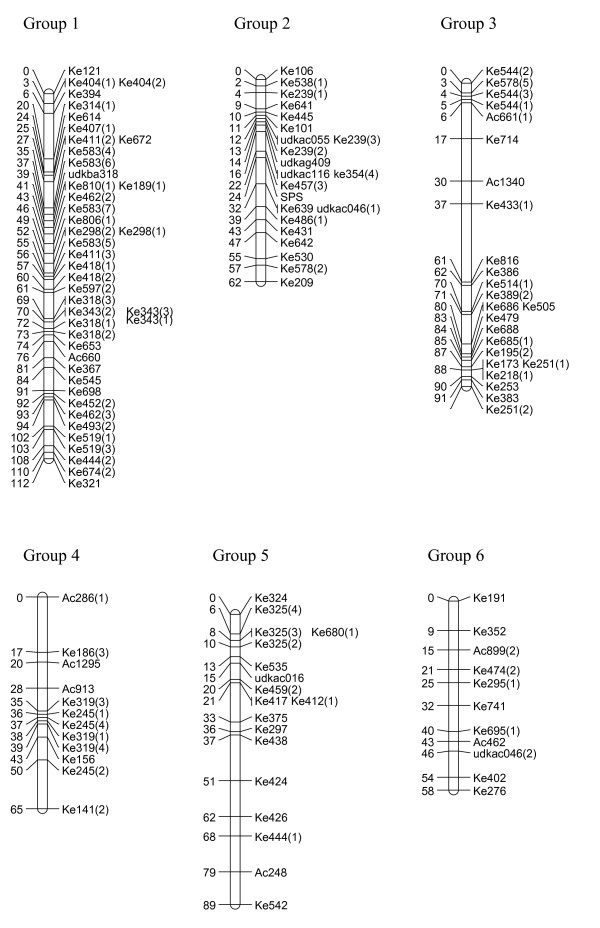
**Genetic linkage map of *Actinidia chinensis *(linkage groups 1–6)**. The markers prefixed 'Ke' were from the kiwifruit EST database and represent expressed genes. Those prefixed 'udk' were from enriched genomic libraries, while all other prefixes relate to the bud libraries, and various markers as described in materials and methods. A number in brackets following a marker name indicates that a single primer pair amplified more than one locus. In the consensus map, 29 linkage groups were defined.

**Figure 2:**
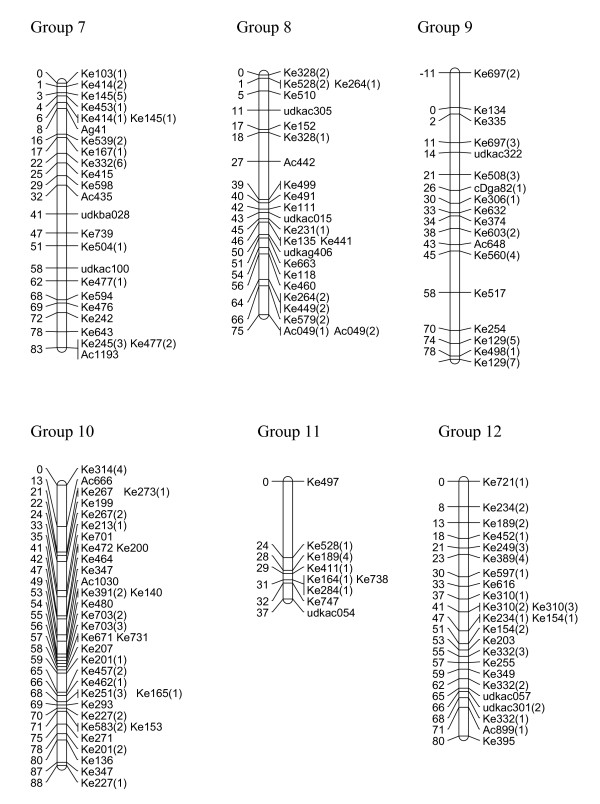
**Genetic linkage map of *Actinidia chinensis *(linkage groups 7–12)**. The markers prefixed 'Ke' were from the kiwifruit EST database and represent expressed genes. Those prefixed 'udk' were from enriched genomic libraries, while all other prefixes relate to the bud libraries, and various markers as described in materials and methods. A number in brackets following a marker name indicates that a single primer pair amplified more than one locus. In the consensus map, 29 linkage groups were defined.

**Figure 3:**
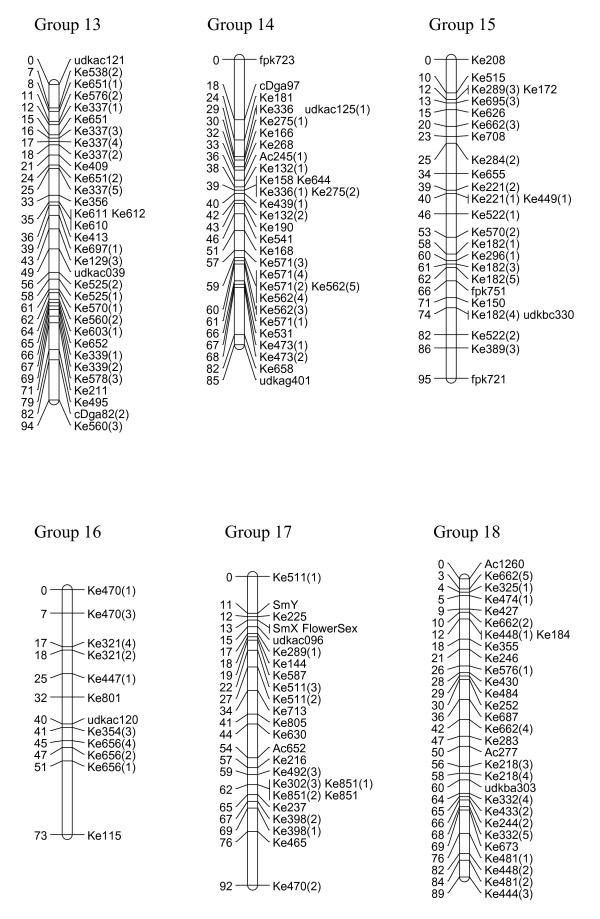
**Genetic linkage map of *Actinidia chinensis *(linkage groups 13–18)**. The markers prefixed 'Ke' were from the kiwifruit EST database and represent expressed genes. Those prefixed 'udk' were from enriched genomic libraries, while all other prefixes relate to the bud libraries, and various markers as described in materials and methods. A number in brackets following a marker name indicates that a single primer pair amplified more than one locus. In the consensus map, 29 linkage groups were defined. Incipient sex chromosomes were identified in Linkage Group 17 where the sex-determining locus was located in the subtelomeric region.

**Figure 4:**
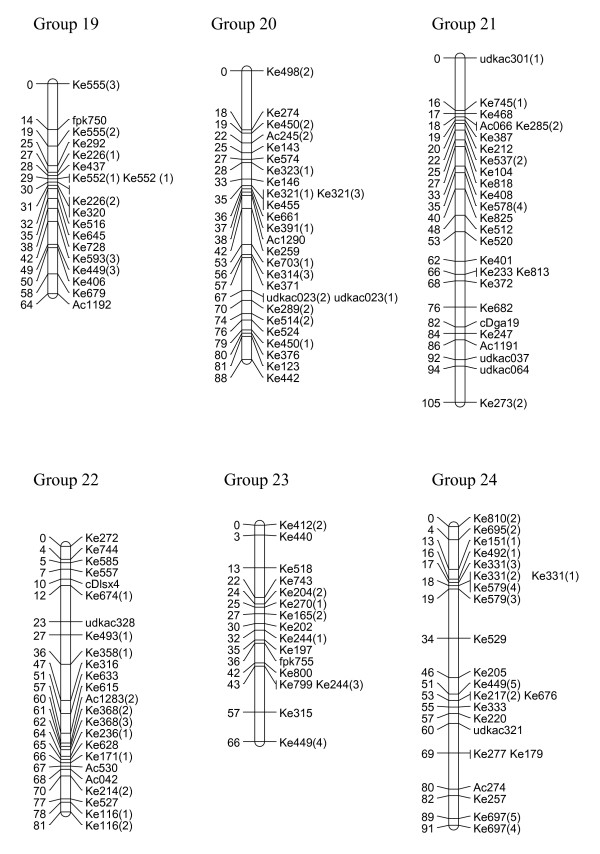
**Genetic linkage map of *Actinidia chinensis *(linkage groups 19–24)**. The markers prefixed 'Ke' were from the kiwifruit EST database and represent expressed genes. Those prefixed 'udk' were from enriched genomic libraries, while all other prefixes relate to the bud libraries, and various markers as described in materials and methods. A number in brackets following a marker name indicates that a single primer pair amplified more than one locus. In the consensus map, 29 linkage groups were defined.

**Figure 5:**
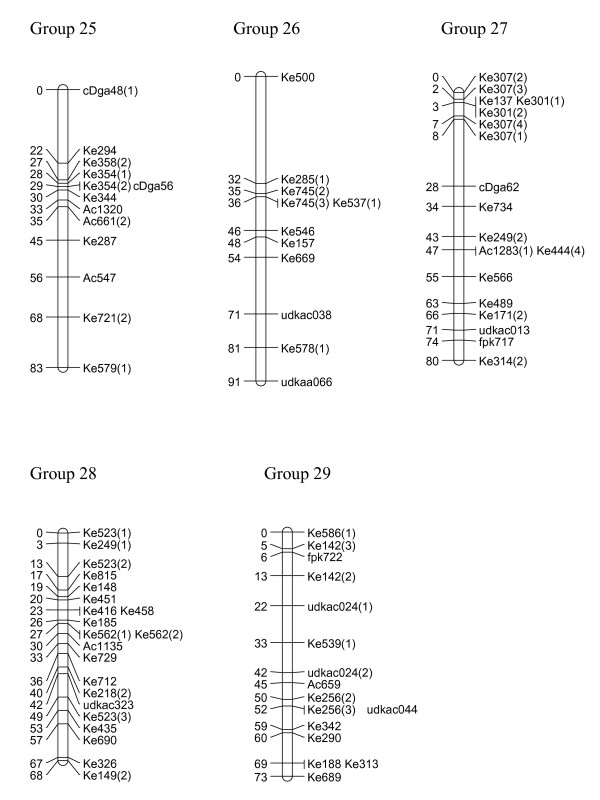
**Genetic linkage map of *Actinidia chinensis *(linkage groups 25–29)**. The markers prefixed 'Ke' were from the kiwifruit EST database and represent expressed genes. Those prefixed 'udk' were from enriched genomic libraries, while all other prefixes relate to the bud libraries, and various markers as described in materials and methods. A number in brackets following a marker name indicates that a single primer pair amplified more than one locus. In the consensus map, 29 linkage groups were defined.

We have created three gene-rich genetic maps, female, male and consensus, identifying the 29 linkage groups of the haploid genome and incipient X and Y sex chromosomes (Figures 1, 2, 3, 4 and 5, Table 1). The female linkage map constructed at LOD 4 and higher, was composed of 464 markers clearly defining 29 linkage groups and covered 2266 cM in the Kosambi function (see Additional file 1). The male map, composed of 365 markers, was shorter than the female map at 2078 cM in length (see Additional file 2). Estimated genome lengths of the female and male parents, using Method 3 of Chakravarti, were 3090 and 2782 cM respectively. The method based on average marker spacing adjustment [32] gave a genome length of 2562 cM in the female and 2402 cM in the male.

**Table 1:** Mapping characteristics of the male and female maps of *A. chinensis*.

Map Characteristic	Male	Female
Total No. of markers	365	464
Total map length (cM)	2078	2266
Range of linkage group length	(20, 123)	(47, 103)
Average marker interval (cM)	6.18	5.12
Marker interval (LQ, Median, UQ)	(1.80, 4.68, 8.66)	(1.41, 3.46, 6.94)
Fully informative markers (%)	222 (60.8)	240 (51.8)
Partly informative markers (%)	13 (3.6)	16 (3.4)
Female informative markers (%)	-	207 (44.6)
Male informative markers (%)	130 (35.6)	-

The same genome lengths with the adjustment for chromosome ends as per Remington *et al*. were 2820 and 2518 cM. A statistical programme that assumes markers are randomly distributed gave an estimate of intra-marker distance. Markers were estimated to be within 10 cM of each other in over 96% and 94% of the female and male genomes respectively (see Additional file 3). These estimates could be somewhat biased due to the non-randomness of marker distribution as noted below. The estimates were based on the adjusted genome lengths. The same genome coverage estimates based on the ratio of observed to estimated genome lengths were 80% and 83% respectively.

In the construction of the male map, Linkage Groups LG11 and LG17 were formatted in two separate sections. However, when the consensus map was compiled, the relatedness of the sections was confirmed through fully informative markers associating them with the corresponding linkage group in the female map. The consensus map, of length 2341 cM, and composed of 636 markers, showed some regions of altered linear order of markers from the sex-related maps (Figures 1, 2, 3, 4 and 5).

The Chi-square value for goodness-of-fit for the female map was 46.5 with 28 *df*. The *p*-value for a larger Chi-square value than observed was 0.015 which indicated a statistically significant departure from a Poisson random process. Linkage groups 1, 10, 14, 18 and 28 had significantly higher number of markers than expected from a random distribution. Similarly, linkage groups 4, 16 and 26 had significantly fewer. For the male map the Chi-square value was 36.6 with 28 *df *and a p-value of 0.128. When related to randomness of marker distribution, this value was non-significant.

### Marker characteristics

A total of 799 primer pairs defining potential microsatellite markers were trialled for map construction. Polymorphism was established between the parents before a marker was evaluated for segregation across the genotypes of 272 siblings in the F_1 _mapping population. A total of 793 SSR markers from the 799 primer pairs tested, the two sex-linked SCAR markers and eight indel/SNP markers were considered to be of sufficient quality to use for map construction. Flower sex phenotype (FlowerSex), with aa alleles in females and ab alleles in males, was mapped as a male informative marker. A number of the markers amplified more than one locus in the mapping population and these are identified by a number in brackets (Figures 1, 2, 3, 4 and 5). Markers that were non-polymorphic in the parents, or non-segregating in the progeny, together made up 39% of the discarded potential markers, 18% of the marker results were difficult to read when one amplifying locus was overlying another and the alleles could not be unequivocally allocated to an individual locus, and 43% showed low information content, non-conforming ratios or poor PCR amplification. In some instances the PCR product was larger than the standard, and unable to be read. This was due to introns amplified from the genomic DNA that were not seen in the EST sequences. The allelic information content of the markers was of four types (Table 2). On the consensus map (Figures 1, 2, 3, 4 and 5) fully informative markers segregated 1:1:1:1 and made up 34% of the markers mapped, female informative and male informative markers segregating 1:1 were 37% and 21% respectively, and 8% were only partly informative segregating 1:2:1 or 3:1. Null alleles featured in all informative groups. Of the 636 markers in the consensus map, 587 markers were EST-derived and representative of expressed genes.

**Table 2:** Allelic information content of microsatellite markers in the *A. chinensis *intraspecific mapping population.

Marker Type	Result from	Parents	Alleles	Segregation	Phenotypes
Fully informative	loci heterozygous	ab × cd	ac ad bc bd	1:1:1:1	4
	in both parents	ab × c0	ac a0 bc b0	1:1:1:1	4
		a0 × bc	ab ac b0 c0	1:1:1:1	4
		ab × ac	aa ac ba bc	1:1:1:1	4
		a0 × b0	ab a0 b0 00	1:1:1:1	4

Partly informative	loci heterozygous	ab × a0	ab 2a b0	1:2:1	3
	in both parents	a0 × ab	ab 2a b0	1:2:1	3
		ab × ab	aa 2ab bb	1:2:1	3
		a0 × a0	3a 00	3:1	2

Female informative	female heterozygous	ab × cc	ac bc	1:1	2
	male homozygous	ab × 00	a0 b0	1:1	2
		a0 × bb	ab b0	1:1	2
		ab × aa	aa ab	1:1	2
		a0 × 00	a0 00	1:1	2

Male informative	male heterozygous	aa × bc	ab ac	1:1	2
	female homozygous	00 × ab	a0 b0	1:1	2
		aa × b0	ab a0	1:1	2
		aa × ab	aa ab	1:1	2
		00 × a0	a0 00	1:1	2

The female alleles were identified first and carried the designation ab if both alleles were visible, the male alleles were cd. Where homology, or null alleles restricted naming the female alleles, then the male alleles were identified in alphabetical order. When null alleles were present, they were informative in various combinations of alleles as shown, but did reduce the information content of the makers overall.

### Identification of the X and Y chromosomes in the female and male linkage maps

The male-sex-linked marker SmY and the phenotype 'FlowerSex' both mapped to a subtelometic region on LG17 in the male genetic map. The fully informative marker udkac096, which mapped in the vicinity of the two markers, was used to identify the corresponding sex linkage group in the female map. While the markers over a large part of the length of these linkage groups were reliably ordered, in a section around the sex markers, some markers were found to alter order after consecutive runs of the map. This unreliably ordered portion of the linkage group contained seven markers including five EST-derived SSRs indicative of expressed genes (Table 3). Primer pairs of these five markers amplified products from both X and Y chromosomes, suggesting conservation of DNA sequence at primer sites on X and Y chromosomes.

**Table 3:** Markers associated with the incipient sex-determining chromosomes in *A. chinensis*.

Marker	Possible gene family
Ke511	Unknown
Ke225	Unknown
udkac096	Dihydroorotate dehydrogenase oxidase
Ke289	Unknown
Ke144	Unknown
Ke587	Zinc finger (C2H2 type) transcription factor family
Ke713	Branching gene
Ke805	Unknown
Ke630	Transducin family protein (WD-40 repeat family protein)
Ac652	Unknown
Ke216	Long-chain acyl-coA synthetase family
Ke492	Abscisic acid-responsive HVA22 family protein
Ke302	Fatty acid omega hydroxylase family
Ke851	Senescence inducible chloroplast stay-green protein
Ke237	Unknown
Ke398	Elongation factor family
Ke465	Unknown
Ke470	Homeobox transcription factor family

The genes associated with individual markers were suggested to belong to certain gene families through BLAST searches.

### Genes mapped to the sex chromosomes

Seventeen markers that mapped to the linkage group containing the sex-determining locus were derived from SSRs associated with ESTs and therefore could be matched to expressed genes. BLAST searches of the GenBANK database were used to try and identify potential functions for these ESTs (Table 3). Nine ESTs showed homology to sequences of known function. However, with the exception of Ke587 and Ke470, with homology to transcription factors, the function of these ESTs appeared unlikely to influence flower sex. The seven remaining genes were all listed in the 'unknown function' category.

## Discussion

### Genetic linkage maps

Gene-rich genetic maps of the *Actinidia chinensis *female and male genomes, together with a consensus map of the two genomes, have been constructed. EST-derived microsatellites proved to be extremely polymorphic and a high proportion were mapped (Table 1). The loci were widely distributed over the 29 linkage groups we have identified. Some linkage groups carried fewer markers than others (Figures 1, 2, 3, 4 and 5) and this may be a reflection of the number of genes contained on a particular linkage group, or may be due to sampling bias in the ESTs selected for mapping. We sampled expressed genes that will be found in euchromatic rather than heterochromatic regions of the genome, not all tissues of the plant were represented, for example, there were no root libraries sampled, and we only selected ESTs that contained a large microsatellite in the transcribed sequence. While our sampling methods will have influenced the non-randomness of the markers on our genetic map, the abundance and relative distribution of microsatellites between transcribed and non-transcribed regions of the genome has been reviewed [33,34], and has been reported to be non-random. Morgante *et al. *[34] report that microsatellite frequency was higher in transcribed regions, particularly in the untranslated portions, than in genomic DNA, and suggest that most microsatellites are found in regions pre-dating the genome expansion in many plants.

Many of the EST-derived primer pairs amplified more than one locus. The loci from a single primer pair could be on separate linkage groups, at a distance from one another on the same linkage group, or, with the resolving power of JoinMap software, unable to be separated on the map. Various authors [7,22,35,36], have considered the possibility that diploid *A. chinensis *is a paleopolyploid, and some evidence they present would support this view. The haploid number of 29 is high, and would suggest that polyploidization may have occurred more than once, and may also have involved hybridization. Alternatively, duplication of a DNA segment may have occurred. This duplication event may have been physically separated by cross-over events or inversions, or tandem repeats may still be in evidence and markers that were unable to be separated could reflect this condition. As one of the primers of a pair was located within the translated portion of the EST to facilitate marker transfer to other species, it is reasonable to suggest that the genes were either duplicated, or members of a gene family with strong sequence homology in the transcribed region. However, a random priming event cannot be ruled out entirely.

### Theoretical putative sex chromosomes

In the genus *Actinidia *all known species are dioecious, so it is reasonable to suggest that dioecy preceded speciation. For the sexes to remain separate over the period of differentiation to speciation, the genes responsible for the male and female characteristics would need to be tightly linked on the two haplotypes of one chromosome, and suppression of recombination would be essential to prevent the recurrence of hermaphroditism. It is not known how many genes are responsible for sex determination. In *Actinidia *we believe there must be at least two genes involved in the development of dioecy. One possible model that has been proposed has a dominant allele for pistil suppression closely linked to a dominant allele for pollen development on the putative Y chromosome, while the equivalent differential segment on the X chromosome has two alleles that function as recessives, one allowing pistil development and one leading to programmed pollen death [4,37].

The genetic structure of the sex-determining region in the genus *Actinidia *has not previously been described. The chromosomes of *Actinidia *are small and of a fairly uniform size, and no definite sex chromosomes have been identified, though He *et al. *[7] described physical characteristics of a theoretical Y chromosome based on cytological studies. The genetic structure of LG 17 would also suggest that, in the male genotype, in a subtelomeric region of the chromosome, recombination was suppressed and marker order was difficult to establish. Lack of recombination is typical of a sex-determining region, so our data support the observation of He *et al. *[7].

There is evidence that sex chromosomes originate from autosomes [38-40], and it is thought that translocation of genetic material to sex chromosomes has occurred [41]. A mutation that produced female or male sterility could, in theory, be found in any part of the chromosome. However, for full dioecy to develop, more than one mutation would need to have occurred, and the sexual differences would need to be fixed in the genome for gender-specific chromosomes to result. Suppression of recombination in the region of the mutations, allowing multiple loci to remain linked, would be required. Such regions of suppressed recombination are known, especially in the heterochromatic regions of the chromosomes such as the pericentromeric and subtelomeric regions of autosomes [10,42]. Pericentromeric locations have been identified as incipient sex-determining loci in asparagus, *Asparagus officinalis *L. [43,44], and papaya, *Carica papaya *[45], both species showing severe suppression of recombination around the sex-determining locus. Again, the recently reported incipient sex chromosomes in the genus *Populus *showed recombination suppression in the vicinity of the gender-linked locus. In *Populus*, like *Actinidia chinensis*, the sex-determining region was identified in the subtelomeric portion of a single chromosome pair [46]. In the sex-determining locus of *Actinidia *several genes were associated with the sex-linked SmX and SmY markers and the phenotype 'FlowerSex'. The suppression of recombination around the sex-determining locus prevents recombination mapping from determining the accurate linear ordering of these genes, and the estimation of their distances from each other.

The genes of unknown function in the sex-determining locus may be similar to unidentified genes in other genera, or they may be specific to floral development in *Actinidia*. The markers have been used to identify bacterial artificial chromosomes (BACs) specific to the region and this is the first step in isolating and characterizing genes of the sex chromosomes in this genus. In addition, the gene-rich linkage map we have constructed will be a valuable resource for quantitative trait loci (QTL) analyses to identify markers related to traits of importance in breeding new and novel kiwifruits for the markets of the world. It will also considerably advance the development of a physical map for map-based cloning of genes for characterization.

## Conclusion

We have described the genetic structure of all 29 linkage groups corresponding to n = 29 in a diploid species of the genus *Actinidia*. Map construction has been robust at LODs between 4 and 10. As the majority of the markers represent expressed genes, we anticipate that this resource will be useful in understanding genetic diversity in the genus. We have also identified, through sex-linked markers, putative X and Y chromosomes. These would appear to be in the early stages of evolution, as the subtelomeric sex-determining locus occupies only a small portion of the chromosome, as assessed through evidence of non-recombination, while the remainder of the chromosomes have the character of autosomes.

## Methods

### Plant material and DNA extraction

An intraspecific mapping population of 272 plants was created in the diploid species *A. chinensis*. Seedlings were screened with the sex marker SmY [5], to ensure equal numbers of female and male plants were propagated. The parents were chosen for their geographic separation in China, the female parent originating from seed from Henan province, Central China, and the male parent from a seed accession from Guangxi province, South China, and for the diversity of fruiting characters which they exhibited, those of the male being inferred from the attributes of female siblings. The mapping population was grown in the Plant and Food research orchard in Te Puke, Bay of Plenty, New Zealand. At budbreak, leaf tissue was taken from each genotype, held at 4° for 24 h, then stored at -80° until required. Seedlings were screened by flow cytometry to ascertain ploidy, and fingerprinted, using a kit of seven previously identified variable microsatellites which occurred in the parents. Only seedlings with the expected genotypes were included in the mapping analysis, all rogue plants were removed. DNA was extracted from young leaf tissue of both parental genotypes and every individual in the mapping population. A sample was ground to powder in liquid nitrogen before being processed through a DNeasy Plant Mini Kit (Qiagen™) according to the manufacturer's instructions. The final eluate was 200 μl in volume. 5 μl of a one in ten dilution of this eluate was used in each PCR reaction.

### Microsatellite identification and primer design

Microsatellites (SSRs) suitable for use as markers were obtained from three sources. The first source was cDNA libraries constructed at Plant and Food Research from floral tissues of *A. chinensis*. The libraries were constructed in the pSport 1 (Not 1-Sal 1) vector and transformed into MAX Efficiency DH5α™ Competent Cells (Life Technologies). Clones were screened for the presence of microsatellite repeats by plaque hybridisation with short (16 bp), ^32^P-labelled probes and positive clones were identified. The second source of SSRs was two genomic libraries enriched either in (AC/GT)_n _or (AG/CT)_n _microsatellite repeats respectively which were constructed at the University of Udine, Italy [35], in Lambda Zap II vector (Stratagene). Transformation into XL1-Blue MRF *Escherichia coli *cells was followed by efficient excision of the plasmid from the Lambda Zap vector by the use of ExAssist^® ^interference-resistant helper phage with SOLR™ cells (Stratagene). DNA from positive clones from both cDNA and genomic libraries was prepared, sequenced, and microsatellites identified. The third source of SSRs was from Plant and Food Research EST databases of *A. chinensis *and *A. deliciosa *sequences [31,47]. Microsatellites from the EST libraries were identified *in silico *as described in Fraser *et al*. [31]. Sequence data from this article have been deposited in the GenBank Data Libraries under accession nos. FG396279–FG528563.

Primer pairs were designed for non-duplicated sequences using the software programme Primer3 (^©^1996, 1997, 1998 [48]). Primer pair sequences were chosen which gave a theoretical PCR product size between 200 and 450 bp, with an annealing temperature between 55° and 60°, and with a GC content of approximately 50%. One of the primers of each pair was located before the microsatellite in the transcribed region, and the other was designed within the translated portion of the EST to facilitate marker transfer to other species. The primer pairs were synthesised and fluorescently-labelled (Dye Sets DS-31 or DS-34) by Applied Biosystems, Australia.

When a microsatellite was not found in the EST database, insertions and deletions (indels) and single nucleotide polymorphisms (SNPs) that were present were used to map particular genes of interest.

### Polymerase chain reaction and electrophoresis

Primer pairs were screened for PCR amplification and length polymorphism with DNA samples of both parents of the mapping population, and the 272 progeny. A reaction mix of 15 μl containing 1 × PCR buffer (20 mM Tris-HCl, 50 mM KCl), MgCl_2 _5 mM (the buffer and MgCl_2 _were those supplied with the polymerase), 0.2 mM each of dNTPs, 4.5 pmol of each primer, and 1.25 units of Platinum Taq polymerase (Invitrogen), was prepared for each DNA sample. About 12.5 ng of genomic DNA was added in 5 μl to bring the total PCR volume to 20 μl. PCRs were performed in a Techne™ TC-412 thermal cycler with a single cycle of 94° for 3 min preceding 35 cycles of denaturing at 94° for 30 sec, annealing for 30 sec, and elongation at 72° for 1 min. PCR reactions were carried out individually before three colour multiplexes of products labelled with 6FAM, TET or HEX (Filter Set C), or 6FAM, VIC or NED (Filter Set D) were prepared for analysis. The allelic content of each genotype was determined by either gel electrophoresis in an ABI PRISM 377 DNA Sequencer (Filter Set C, TAMRA™ size standard), and analyzed with GeneScan Analysis and Genotyper software (Applied Biosystems), or capillary electrophoresis in an ABI Prism^® ^3100 Genetic Analyzer (Filter Set D, ROX™ size standard), and analyzed with GeneMapper™ Software Version 3.0 (Applied Biosystems). All markers were scored by at least two people independently for verification.

### Data analysis and map construction

Chi-square tests of goodness-of-fit to expected segregation ratios of 1:1:1:1, 1:2:1, 3:1 or 1:1 were carried out for all markers segregating in the F_1 _progeny. The two sex-linked SCAR markers SmX and SmY previously developed in *A. chinensis *[5], were scored in the same fashion. The sex of the flowers of all genotypes in the mapping population was scored, and this phenotype was mapped as the 'FlowerSex' locus. Female, male and consensus linkage maps were constructed for the F_1 _progeny using JoinMap^® ^3.0 [49]. For grouping of marker loci Joinmap uses a minimum LOD score only. A range of LOD scores, 4 – 10, were used so that linkage groups that were consistent across the range could be identified. In JoinMap, linkage is considered transitive, i.e. if A is linked to B which is linked to C, then A, B and C are linked. This gives one grouping of markers for each of the given threshold values. JoinMap uses only two-point (pairwise) analysis for ordering of markers. There are other software packages that use multi-point likelihood methods. One such package OutMap [50], which is also designed for out-crossing species, was used to validate a sample linkage group for consistency in marker order and distances. For ordering of markers within a linkage group, JoinMap uses a sequential build-up starting with the most informative, and 'rippling' for local optima. Map distances are estimated by minimising the sum of weighted (LOD scores as weights), squared difference of observed and expected pairwise distances [51].

The observed total map length was calculated simply as the sum of map distances of the terminal markers of each linkage group. Several methods were then used to provide estimates of the total genetic length of the genome. Firstly, the genome length was estimated using Method 3 of Chakravarti *et al. *[52], which is a modification of the method-of-moments estimator proposed by Hulbert *et al. *[53]. This method estimates the genome length by multiplying, the ratio of total marker pairs to the number of marker pairs that equals or exceeds a specified LOD threshold value, by the map distance corresponding to the largest observed recombination fraction among the latter marker pairs. A threshold LOD value of 7 and the Kosambi genetic distance were used [54]. The second method used was an adjustment to the Hulbert's method proposed by Remington *et al*. [32] to correct for the upward bias due to ends of linkage groups. The adjustment is given as

$$\hat{L}=\frac{n(n-1)d}{2k}\left[1+{\left(1-\frac{2Ck}{n(n-1)}\right)}^{1/2}\right]$$

where *n *is the total number of markers, *k *is the number of marker pairs having a LOD equal to, or greater than, the specified threshold value, *d *is the map distance corresponding to the above LOD threshold and *C *is the haploid chromosome number. A third method used estimated individual linkage group length as the observed length plus twice the average marker spacing. This is based on the assumption that marker position is uniformly distributed, and the expected distance from a terminal marker to chromosome end is then equal to the average marker spacing [32]. Adding these individual linkage group lengths gave an estimate of the total genome length.

Genome coverage was estimated first as the proportion of observed total map length to the estimated genome length. A second method used the following equation proposed by Lange and Boehnke [55], which makes the assumption markers are randomly distributed throughout the genome

*c *= 1 - *e*^-2*dn*/*L*^

where *c *is the proportion of genome within *d *cM of a marker, *n *and *L *are the number of markers and the estimated genome length.

Distribution of markers among linkage groups was assessed by comparing observed marker numbers in linkage groups with expectations under a random Poisson process. The expected number of markers for linkage group *i *under the null hypothesis is given by the Poisson parameter *λ*_*i *_= *nL*_*i*_/∑ *L*_*i*_, where *L*_*i*_s are lengths estimated by the third method described above. A chi-square value for goodness-of-fit was computed as ∑ [(*O*_*i *_- *E*_*i*_)^2^/*E*_*i*_], where *O *and *E *are the observed and expected values, and the corresponding degrees of freedom is equal to one less than the number of linkage groups.

## Authors' contributions

LGF conceived the study, carried out SSR discovery, marker polymorphism discovery and population genotyping, and drafted the manuscript. GKT, CFH, and GPG performed SSR and marker polymorphism discovery and population genotyping, GKT also assisted in preparing the manuscript. PMD performed genotyping analyses and map construction. MAM recommended the most suitable mapping population and performed genotyping analyses. HNDS assisted with map construction, carried out all statistical analyses and wrote the statistical methods section for the manuscript. RNC developed the bioinformatics system used for SSR discovery. All authors read and approved the final manuscript.

## Supplementary Material

Additional file 1 **Genetic linkage map (female) of *Actinidia chinensis*.** The markers prefixed 'Ke' were from the kiwifruit EST database and represent expressed genes. Those prefixed 'udk' were from enriched genomic libraries, while all other prefixes relate to the bud libraries, and various markers as described in materials and methods. A number in brackets following a marker name indicates that a single primer pair amplified more than one locus. In the female map 29 linkage groups were defined. Incipient sex chromosomes were identified in Linkage Group 17 where the sex-determining locus was located in the subtelomeric region. Click here for file

Additional file 2 **Genetic linkage map (male) of *Actinidia chinensis*.** The markers prefixed 'Ke' were from the kiwifruit EST database and represent expressed genes. Those prefixed 'udk' were from enriched genomic libraries, while all other prefixes relate to the bud libraries, and various markers as described in materials and methods. A number in brackets following a marker name indicates that a single primer pair amplified more than one locus. In the male map 29 linkage groups were defined. Incipient sex chromosomes were identified in Linkage Group 17 where the sex-determining locus was located in the subtelomeric region. Click here for file

Additional file 3 **Marker distribution in the female and male maps.** A statistical programme that assumes markers are randomly distributed gave an estimate of intra-marker distance. Markers were estimated to be within 10 cM of each other in over 96% and 94% of the female and male genomes respectively. Click here for file
