# Supplementary material for: A gene-rich linkage map in the dioecious species Actinidia chinensis (kiwifruit) reveals putative X/Y sex-determining chromosomes
Source: BMC Genomics. 2009 Mar 10;10:102. doi: 10.1186/1471-2164-10-102 (PMC2661093; doi:10.1186/1471-2164-10-102)
Supplement: Additional file 1 — Genetic linkage map (female) of Actinidia chinensis. The markers prefixed 'Ke' were from the kiwifruit EST database and represent expressed genes. Those prefixed 'udk' were from enriched genomic libraries, while all other prefixes relate to the bud libraries, and various markers as described in materials and methods. A number in brackets following a marker name indicates that a single primer pair amplified more than one locus. In the female map 29 linkage groups were defined. Incipient sex chromosomes were identified in Linkage Group 17 where the sex-determining locus was located in the subtelomeric region. [file 1471-2164-10-102-S1.doc]

Group 1 Group 2 Group 3

Ke544(2)

0

Ke578(5)

3

Ac661(1)

7

Ke714

20

Ac1340

30

Ke433(1)

34

Ke201(2)

48

Ke386

58

Ke389(2)

63

Ke514(1)

64

Ke686

73

Ke479

74

Ke688

76

Ke685(1)

78

Ke251(1)

79

Ke173

80

Ke218(1)

81

Ke195(2)

82

Ke251(2)

83

Ke383

85

Ke253

86

Ke404(2)

Ke404(1)

0

Ke394

3

Ke121

6

udkba318

18

Ke672

22

Ke407(1)

23

Ke204(3)

26

Ke614

32

Ke583(4)

Ke583(6)

34

Ke411(3)

Ke411(2)

37

Ke597(2)

52

Ke343(2)

Ke343(1)

56

Ac660

63

Ke367

69

Ke698

74

Ke462(3)

Ke452(2)

75

Ke493(2)

76

Ke545

82

Ke519(3)

87

Ke519(1)

88

Ke444(2)

91

Ke674(2)

93

Ke239(1)

0

Ke538(1)

3

Ke106

6

Ke641

8

udkag409

11

udkac116

13

udkac055

15

Ke101

16

Ke457(3)

22

SPS

27

Ke639

30

Ke486(1)

37

Ke642

44

Ke530

50

Ke578(2)

52

Ke209

53

Group 4 Group 5 Group 6

Ke324

0

Ke325(3)

9

Ke535

12

udkac016

15

Ke412(1)

20

Ke417

21

Ke297

33

Ke438

34

Ke444(1)

59

Ke542

71

Ac248

82

58

Ac286(1)

0

Ac1295

17

Ac913

24

Ke319(4)

32

Ke319(1)

34

Ke141(2)

Ke191

0

Ke352

14

Ac899(2)

20

Ke474(2)

26

Ke295(1)

29

Ke741

36

Ke695(1)

44

udkac046(2)

47

Ac462

51

Ke576(3)

58

Ke276

76

Group 7 Group 8 Group 9

Ke145(1)

Ke145(5)

0

Ke414(1)

Ke453(1)

1

Ag41

4

Ke539(2)

12

udkba028

34

Ke739

38

udkac100

50

Ke477(2)

Ke594

57

Ke476

Ke477(1)

59

Ke242

63

Ke245(3)

74

Ke368(1)

0

Ke663

3

Ke510

28

Ac442

31

Ke328(2)

Ke264

34

Ke152

38

Ke328(1)

44

udkac305

45

udkac015

Ke499

56

Ke491

57

Ke111

58

Ke441

Ke231(1)

61

Ke135

62

udkag406

66

Ke118

69

Ke460

73

Ke449(2)

81

Ac049(1)

89

Ac049(2)

90

Ke134

0

Ke335

3

udkac322

7

Ke697(3)

13

Ke508(3)

19

cDga82(1)

22

Ke632

30

Ke374

31

Ke603(2)

35

Ac648

41

Ke560(4)

42

Ke517

55

Ke254

69

Ke129(5)

71

Ke498(1)

73

Ke129(7)

76

Group 10 Group 11 Group 12

Ke213(1)

0

Ac666

10

Ke199

19

Ke273(1)

20

Ke701

28

Ke472

30

Ke200

32

Ac1030

Ke347

38

Ke140

42

Ke703(2)

44

Ke731

Ke671

45

Ke207

46

Ke201(1)

47

Ke165(1)

51

Ke462(1)

53

Ke457(2)

54

Ke251(3)

55

Ke227(2)

Ke153

58

Ke271

60

Ke136

65

Ke227(1)

75

Ke581

84

Ke497

0

Ke528(1)

24

Ke189(4)

28

Ke411(1)

29

Ke164(1)

Ke738

31

Ke284

Ke747

32

udkac054

37

Ke175

71

Ac212

75

Ke806(2)

81

Ke251(4)

83

Ke251(2)

88

Ke721(1)

0

Ke234(2)

6

Ke189(2)

13

Ke249(3)

16

Ke389(4)

26

Ke616

39

Ke310(2)

Ke310(3)

46

Ke255

62

udkac057

71

Ac899(1)

73

Ke332(1)

76

Ke332(2)

77

Ke395

81

Group 13 Group 14 Group 15

fpk723

0

Ke446(1)

10

Ke181

22

Ke336

27

Ke275(1)

udkac125(1)

28

Ke166

30

Ke439(1)

Ke336(1)

Ke275 (2)

34

Ke644

35

Ac245(1)

38

Ke158

39

Ke132(1)

40

Ke268

41

Ke132(2)

43

Ke541

46

Ke168

52

cDga97

56

Ke571(4)

60

Ke562(4)

Ke562(3)

Ke571(2)

61

Ke571(1)

63

Ke531

68

Ke473(1)

69

Ke473(2)

70

Ke658

82

udkag401

84

udkac121

0

Ke538(2)

6

Ke337(1)

12

Ke651

Ke651(2)

16

Ke409

19

Ke337(4)

23

Ke560(3)

25

Ke337(5)

26

Ke413

34

udkac039

46

Ke525(2)

53

Ke525(1)

54

Ke570(1)

58

Ke339(2)

59

Ke603(1)

60

Ke652

62

Ke339(1)

64

Ke211

73

cDga82(2)

81

Ke289(3)

Ke172

0

Ke515

Ke695(3)

2

Ke626

3

Ke655

21

Ke221(1)

Ke221(2)

28

Ke296(1)

47

Ke182(3)

48

fpk751

54

Ke150

60

Ke182(1)

67

fpk721

69

Ke389(3)

73

Ke522(2)

79

udkbc330

80

Ke522(1)

103

Group 16 Group 17 Group 18

Ke511(3)

0

Ke587

3

Ke144

4

udkac096

7

Ke289(1)

8

Ke713

22

Ke492(3)

35

Ac652

42

Ke216

43

Ke302(3)

47

Ke237

50

Ke398(1)

54

Ke398(2)

55

Ke465

63

Ke492(2)

76

Ke470(2)

79

Ac1260

0

Ke325(1)

2

Ke474(1)

4

Ke427

5

Ke662(2)

8

Ke184

9

Ke246

18

Ke252

25

Ke147(2)

26

Ke484

30

Ke662(4)

36

Ke283

44

Ac277

47

Ke218(3)

51

Ke218(4)

52

udkba303

53

Ke433(2)

Ke244(2)

58

Ke332(5)

61

Ke673

62

Ke481(1)

67

Ke448(2)

77

udkac120

0

Ke522(3)

9

Ke321(4)

33

Ke447(1)

41

Ke656(4)

51

Ke801

63

Group 19 Group 20 Group 21

Ke498(2)

0

Ke274

18

Ac1290

29

Ke143

34

Ke146

40

Ke442

57

Ke123

64

Ke376

65

Ke450(1)

66

Ke524

Ke514(2)

71

Ke289(2)

76

udkac023(1)

78

udkac023(2)

79

Ke371

84

Ke314(3)

89

Ac1191

0

udkac301

15

Ke285(2)

22

Ke537(2)

29

Ke745(1)

Ke468

30

Ac066

31

Ke212

Ke387

32

Ke818

39

Ke578(4)

Ke408

47

cDga19

57

Ke512

63

Ke520

67

Ke401

74

Ke233

78

Ke813

79

Ke372

80

Ke682

89

Ke247

95

udkac037

102

Ke593(3)

0

Ke406

11

Ke320

15

Ke226(1) Ke292

16

Ke728

24

fpk750

31

Ke679

47

Group 22 Group 23 Group 24

Ke272

0

Ke744

4

cDlsx4

8

Ke557

11

udkac328

21

Ke358(1)

32

Ke316

39

Ac1283(2)

49

Ac042

53

Ac530

54

Ke171(1)

55

Ke628

57

Ke368(3)

58

Ke615

59

Ke214(2)

61

Ke116(1)

64

Ke527

66

Ke116(2)

70

Ke683

0

Ke412(2)

33

Ke440

36

Ke518

46

Ke244(1)

48

Ke197

52

Ke202

54

Ke270(1)

55

Ke743

57

Ke204(2)

60

Ke165(2)

61

fpk755

71

Ke331(1)

0

Ke579(3)

1

Ke492(1)

10

Ke529

19

udkac321

35

Ke333

42

Ke676

44

Ke449(5)

47

Ke220

48

Ke205

52

Ke277

57

Ac274

66

Ke257

70

Ke697(5)

76

Group 25 Group 26 Group 27

cDga48(1)

0

Ke248(1)

16

Ke294

20

Ke656(3)

23

Ke358(2)

24

Ke344

27

Ac1320

30

Ke721(2)

34

Ke579(1)

49

Ke287

60

Ac547

71

cDga56

79

Ac661(2)

87

Ke500

0

Ke217(1)

15

Ke285(1)

33

Ke745(2)

35

Ke745(3)

Ke537(1)

36

Ke546

49

Ke157

51

Ke669

59

udkac038

77

udkaa066

97

Ke307(3)

0

cDga62

11

Ke444(4)

26

Ke249(2)

30

Ke566

39

Ac077

42

Ke489

46

Ke171(2)

50

fpk717

57

Group 28 Group 29

Ke249(1)

0

Ke523(2)

Ke523(1)

2

Ke451

14

Ke148

Ke815

15

Ke416

Ke458

19

Ke562(2)

23

Ke185

25

Ac1135

26

Ke729

29

Ke712

32

Ke218(2)

36

udkac323

38

Ke435

49

Ke690

53

Ke326

Ke149(2)

64

Ke142(2)

0

Ke142(3)

4

fpk722

15

Ke586(1)

19

Ke539(1)

29

Ac659

42

udkac024(1)

44

udkac044

58

Ke342

Ke290

65

Ke188

69

Ke689

71

Ke313

78
