# Supplementary material for: A gene-rich linkage map in the dioecious species Actinidia chinensis (kiwifruit) reveals putative X/Y sex-determining chromosomes
Source: BMC Genomics. 2009 Mar 10;10:102. doi: 10.1186/1471-2164-10-102 (PMC2661093; doi:10.1186/1471-2164-10-102)
Supplement: Additional file 2 — Genetic linkage map (male) of Actinidia chinensis. The markers prefixed 'Ke' were from the kiwifruit EST database and represent expressed genes. Those prefixed 'udk' were from enriched genomic libraries, while all other prefixes relate to the bud libraries, and various markers as described in materials and methods. A number in brackets following a marker name indicates that a single primer pair amplified more than one locus. In the male map 29 linkage groups were defined. Incipient sex chromosomes were identified in Linkage Group 17 where the sex-determining locus was located in the subtelomeric region. [file 1471-2164-10-102-S2.doc]

Group 1 Group 2 Group 3

Ke404(2)

Ke404(1)

0

Ke121

2

Ke314(1)

14

Ke411(2)

17

Ke614

22

udkba318

31

Ke810(1)

35

Ke806(1)

45

Ke298(2) Ke298 (1)

48

Ke418(1)

52

Ke418(2)

55

Ke318(3)

65

Ke343(3)

66

Ke343(2)

67

Ke318(1)

68

Ke318(2)

69

Ke653

70

Ac660

72

Ke367

76

Ke545

81

Ke698

86

Ke493(2)

91

Ke519(1)

96

Ke519(3)

98

Ke674(2)

106

Ke321

109

Ke106

0

udkac116

14

Ke239(2)

16

ke354(4)

18

Ke641

20

Ke445

22

SPS

28

udkac046(1)

38

Ke639

39

Ke486(1)

46

Ke431

51

Ke642

56

Ke530

66

Ke209

81

Ke544(1)

0

Ke714

8

Ac1340

23

Ke383

35

Ke433(1)

37

Ke173

Ke253

38

Ke685(1)

Ke688

39

Ke479

40

Ke505

44

Ke386

Ke816

70

Group 4 Group 5 Group 6

Ac286(1)

0

Ke186(3)

20

Ac1295

23

Ac913

31

Ke245(2)

35

Ke319(4)

40

Ke319(3)

41

Ke245(1)

42

Ke245(4)

43

Ke156

49

Ke325(3)

0

Ke325(4)

2

udkac016

12

Ke459(2)

16

Ke417

Ke412(1)

17

Ke375

30

Ke297

Ke438

35

Ke424

49

Ke426

59

Ac248

81

Ke352

0

Ke741

26

Ke402

35

Ke276

36

udkac046(2)

43

Group 7 Group 8 Group 9

Ke145(5)

0

Ke453(1)

3

Ke145(1)

5

Ke414(1)

6

Ke414(2)

8

Ke167(1)

19

Ke332(6)

25

Ke415

27

Ke598

31

Ac435

34

udkba028

44

Ke504(1)

54

udkac100

61

Ke477(1)

73

Ke643

83

Ac1193

87

Ke335

0

Ke697(2)

23

udkac322

43

Ke306(1)

61

Ke632

Ke374

65

Ac648

72

Ke254

98

Ke579(1)

100

Ke498(1)

108

Ke509

118

Ke129(6)

123

Ac442

0

udkac015

18

udkag406

22

Ke441

24

Ke118

29

Ke328(2)

38

Ke264

40

Ke328(1)

43

Ke579(2)

44

udkac305

49

Ac049(1)

53

Ac049(2)

54

Ke528(2)

59

Group 10 Group 11 Group 12

Ke314(4)

0

Ke213(1)

10

ac666

16

Ke273(1)

Ke199

21

Ke701

40

Ke200

47

Ke464

Ke472

50

Ke347

55

ac1030

59

Ke480

62

Ke201(2)

65

Ke201(1)

68

Ke731

71

Ke583(2)

81

Ke164(1)

Ke738

0

Ke747

Ke284(1)

1

udkac054

7

Ke806(2)

0

Ke222

2

Ke251(3)

4

Ke685(2)

10

Ke496

14

Ke251(2)

16

Ac286(2)

21

Ke203

0

Ke234(1)

10

Ke349

14

Ke249(3)

19

Ke616

29

Ke310(1)

37

Ke234(4)

38

udkac301

42

Ke154(1)

55

Ke154(2)

56

Ke332(3)

60

Ke332(2)

64

udkac301(2)

69

udkac057

70

ac899(1)

78

Ke395

90

Group 13 Group 14 Group 15

udkac121

0

Ke651(1)

16

Ke651(2)

17

Ke576(2)

19

Ke337(3)

Ke337(4)

25

Ke337(5)

30

Ke356

40

Ke611

Ke612

42

Ke610

43

Ke697(1)

46

Ke129(3)

50

udkac039

57

Ke560(2)

Ke339(1)

69

Ke339(2)

Ke652

Ke603(1)

72

Ke211

74

Ke578(3)

75

Ke495

86

cDga82(2)

87

Ke644

0

Ke439(1)

2

Ke190

4

Ke168

10

Ke571(3)

15

Ke571(2)

Ke562(4)

Ke562(5)

17

Ke531

25

Ke658

42

udkag401

47

Ke515

0

Ke208

Ke626

8

Ke662(3)

15

Ke284(2)

21

Ke655

33

Ke449(1)

39

Ke570(2)

52

Ke182(1)

59

Ke182(5)

61

udkac330

67

Ke150

71

Ke389(3)

86

fpk721

95

Group 16 Group 17 Group 18

udkac120

0

Ke470(1)

23

Ke470(3)

28

Ke321(2)

40

Ke801

58

Ke656(1)

76

Ke511(2)

0

udkac096

12

FlowerSex

SmX

14

Ke225

15

SmY

16

Ke630

40

Ke216

0

Ke302(3)

4

Ke237

Ke398(2)

6

Ke470(2)

30

Ac1260

0

Ke474(1)

8

Ke427

15

Ke430

42

Ke252

44

Ke283

59

Ac277

61

Ke218(3)

68

Ke218(4)

70

udkba303

75

Ke332(4)

79

Ke673

86

Ke481(1)

95

Ke481(2)

101

Group 19 Group 20 Group 21

Ke552(2) Ke552 (1)

0

Ke226(2)

1

Ke406

5

Ke555(2)

11

Ke555(3)

29

Ke679

45

fpk750

51

Ke292

58

Ke226(1)

59

Ke437

61

Ke516

64

Ke645

67

Ke728

72

udkac024(1)

75

Ke449(3)

85

Ac1192

99

Ke274

0

Ac245(2)

5

Ac1290

24

Ke123

43

Ke524

46

udkac023(1)

55

udkac023(2)

56

Ke703(1)

67

Ke371

73

Ke259

82

Ke661

86

Ke455

87

Ke391(1)

88

Ke323(1)

96

Ke574

98

Ke450(2)

105

Ke825

0

Ke520

10

Ke512

14

Ke401

23

Ke233

29

Ke408

36

Ke247

47

udkac037

56

udkac064

58

Ke273(2)

68

Group 22 Group 23 Group 24

Ke585

0

Ke557

1

udkac328

22

Ke493(1)

27

Ke358(1)

36

Ke316

50

Ke633

52

Ke615

57

Ke368(2)

63

Ke236(1)

66

Ke628

67

Ke171(1)

69

Ac530

70

Ke527

82

Ke116(2)

85

Ke116(1)

86

Ke244(1)

0

Ke799

Ke244(3)

Ke800

14

Ke518

18

Ke270(1)

23

Ke315

28

fpk755

32

Ke449(4)

39

Ke810(2)

0

Ke695(2)

5

Ke151(1)

14

Ke331(3)

16

Ke579(4)

18

Ke529

34

Ke583(1)

44

udkac321

49

Ke333

54

Ke179

65

Ke220

70

Ac274

Ke217(2)

76

Ke205

86

Group 25 Group 26 Group 27

cDga48(1)

0

Ke354(1)

Ke358(2)

27

Ke344

30

ac1320

33

Ke294

39

Ke157

0

udkac038

16

udkaa066

36

Ke307(1)

0

Ke307(4)

1

Ke301(2)

Ke301(1)

5

cDga62

28

Ke734

33

Ke249(2)

41

ac1283(1)

46

Ke566

55

Ke489

65

udkac013

72

Ke314(2)

80

Group 28 Group 29

Ke185

0

Ke562(1)

7

Ke729

14

Ke712

16

udkac323

20

Ac659

0

udkac024(2)

3

Ke256(3)

7

Ke256(2)

9

Ke648

12

Ke342

16

Ke290

17

Ke473(3)

20

Ke689

37

Ke188

43
