# Supplementary figures and images for: A gene-rich linkage map in the dioecious species Actinidia chinensis (kiwifruit) reveals putative X/Y sex-determining chromosomes
Source: BMC Genomics. 2009 Mar 10;10:102. doi: 10.1186/1471-2164-10-102 (PMC2661093; doi:10.1186/1471-2164-10-102)

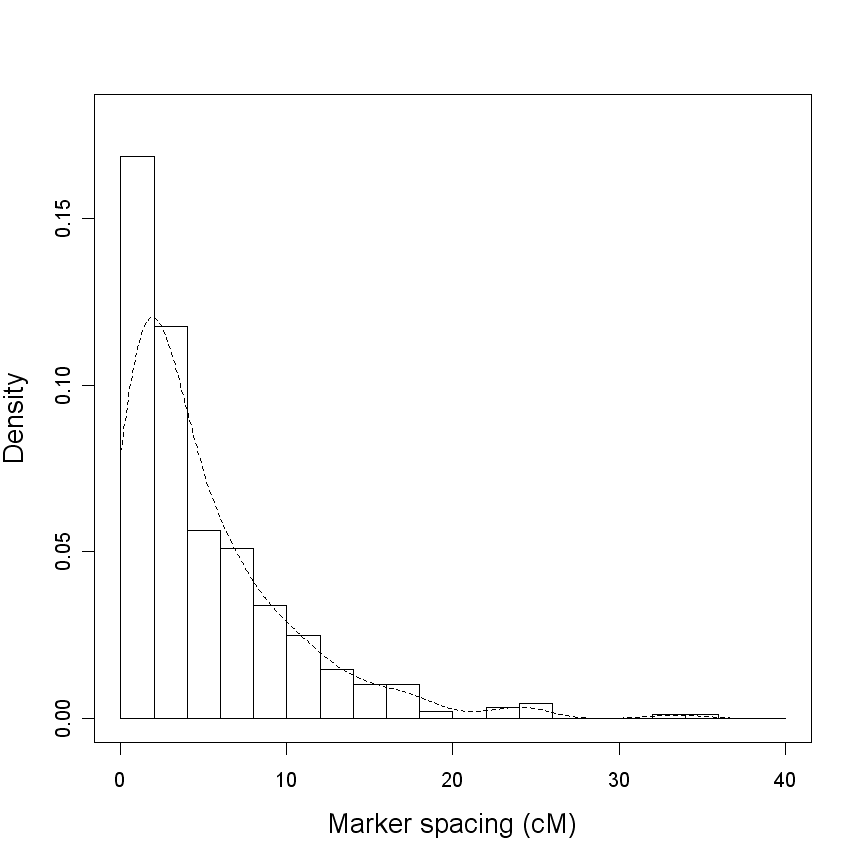

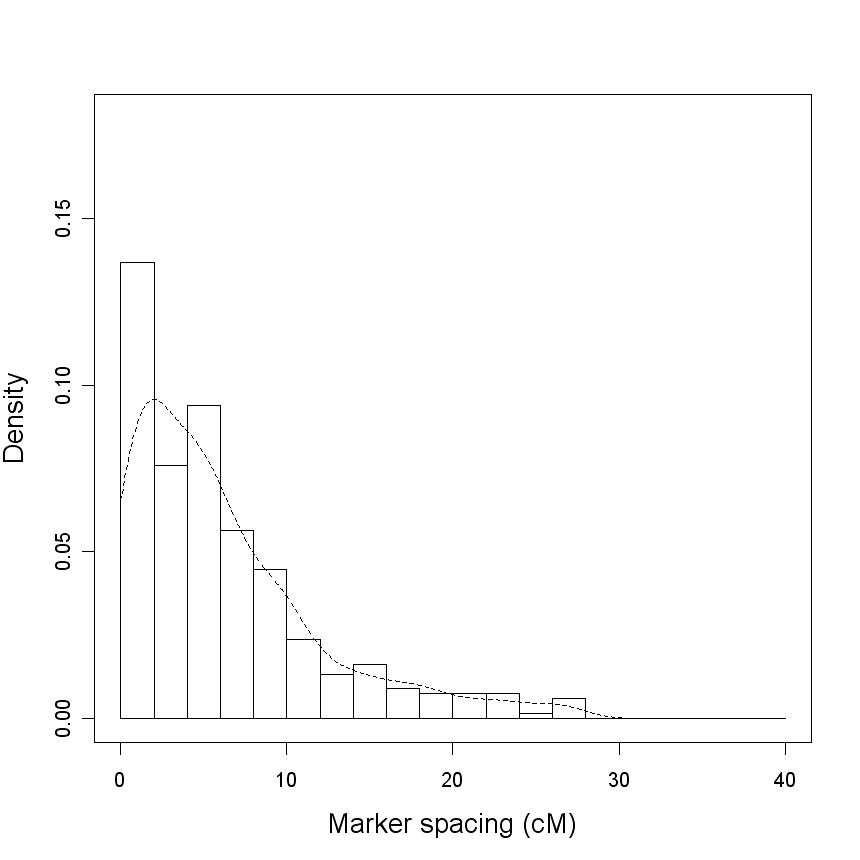


Female Male

Supplement: Additional file 3 — Marker distribution in the female and male maps. A statistical programme that assumes markers are randomly distributed gave an estimate of intra-marker distance. Markers were estimated to be within 10 cM of each other in over 96% and 94% of the female and male genomes respectively. [file 1471-2164-10-102-S3.doc]
